# Decoding phage communication: molecular networks, evolutionary dynamics, and therapeutic applications

**DOI:** 10.1038/s41522-026-01050-3

**Published:** 2026-06-12

**Authors:** Sahar Abed, Mohammad Sholeh, Sara Sadeghi, Ameneh Khatami, Peter Speck, Morvarid Shafiei

**Affiliations:** 1 Department of Microbial Biotechnology, Faculty of Basic Sciences and Advanced Technologies in Biology, University of Science and Culture, Tehran, Iran; 2 Department of Bacteriology, Pasteur Institute of Iran, Tehran, Iran; 3 Department of Biological Sciences, Idaho State University, Pocatello, ID USA; 4 Sydney Infectious Diseases Institute, University of Sydney, Camperdown, NSW Australia; 5 Department of Infectious Diseases and Microbiology, The Children’s Hospital at Westmead, Sydney, NSW Australia; 6 College of Science and Engineering, Flinders University, Bedford Park, SA Australia

**Keywords:** Biological techniques, Biotechnology, Microbiology

## Abstract

Communication between bacteriophages, particularly in biofilms, has long been studied. The recent discovery of the arbitrium lysis-lysogeny switch in Bacillus phages, similar to bacterial quorum sensing, has renewed interest in phage communication. This review examines the arbitrium system alongside other switching mechanisms, explores its role in pathogen-phage-host immune interactions, and proposes design principles for “smart” phage therapies.

## Introduction

Bacteriophages (“phages”) are viruses that infect bacteria, representing the most abundant biological entities on Earth. Global estimates suggest that phage particles number approximately 10^31^, outnumbering their prokaryotic hosts by at least an order of magnitude in most environments^1^. In marine systems, for instance, phage-induced bacterial mortality can daily account for 20–40% of the standing microbial stocks, underscoring their pivotal role in constraining microbial population sizes and driving community turnover. Such pervasive predation exerts top-down control that shapes microbial diversity through “kill-the-winner” dynamics, wherein phages preferentially infect the most abundant bacterial lineages, thereby preventing competitive exclusion and promoting coexistence^1^.

Phages exhibit two primary reproductive strategies: lytic and lysogenic cycles, which underpin their ecological functions. In the lytic cycle, virulent phages commandeer host metabolism to produce progeny, ultimately leading to host cell lysis and release of viral particles and organic cellular debris into the environment^2–4^. Conversely, temperate phages can integrate their genomes into bacterial chromosomes as prophages, modulating host physiology and fitness in ways that include contributing accessory genes and conferring immunity to superinfection by related phages^2^. These integrated elements not only influence host gene expression but also serve as reservoirs for auxiliary metabolic genes, which can be maintained and transferred among microbial taxa, expanding the collective metabolic repertoire of microbial communities^1^.

The ecological impacts of phage-mediated lysis extend beyond direct host mortality. Virus-induced lysis liberates dissolved organic matter and inorganic nutrients, such as nitrogen and phosphorus, which can be recycled by surrounding microbes in a process termed the “viral shunt”^5^. In soil ecosystems, for example, phage lysis has been linked to increased ammonium (NH_4_^+^) concentrations, suggesting that phages can directly influence nutrient turnover and the availability of plant-accessible nitrogen pools^6^. In aquatic environments, phage predation on dominant bacterial clades, including the Alphaproteobacteria clade SAR11, affects not only host population dynamics but also global biogeochemical cycles by influencing carbon export from surface waters and production of climatically active gases^7^.

Phages operate across diverse habitats, from hyperarid desert soils to the human gastrointestinal tract, and their interactions with bacterial hosts are context-dependent. In the former, microscale heterogeneity dictates phage–host encounter rates, influencing phage distribution and diversity^8,9^. Within the plant phyllosphere, phage predation modulates bacterial abundance and community composition, suggesting potential applications for promoting plant health and resilience^10^. In the human gut, phages form a dynamic community of lytic and temperate viruses that drive bacterial diversification, horizontal gene transfer, and ecosystem stability, with direct implications for host health and disease^2^.

Beyond direct predation, phages interact with other mobile genetic elements and microbial components to shape community assembly and function. The coexistence of phages with plasmids and transposons can lead to either antagonistic or synergistic effects on bacterial diversity, influencing overall community composition and stability^11^. In synthetic and natural microbial consortia, phage–bacteria interactions can indirectly affect metabolic cross-feeding and niche partitioning, preventing dominance by any single taxon and fostering biodiversity and resilience^12–14^. Collectively, these multifaceted roles of phages as predators, gene vectors, and modulators of nutrient cycling underscore their importance as key evolutionary and ecological engineers in microbial ecosystems.

Phage communication encompasses the capacity of viruses to produce, release, and detect signals, including specific molecular signals, thereby coordinating collective behaviors within viral populations. Evidence of phage communication is almost as old as the early studies of phage biology, with the observation of “lysis inhibition” (LIN) arising out of work by Doermann^15^ and Hershey^16^ in the 1940s. In this phenomenon, phages of *Escherichia coli* considered exclusively lytic are inhibited in their lytic behavior by superinfection with other phages, delaying lysis and boosting progeny yield. Multiple phage genes and gene products, and mechanisms, appear to be involved in LIN, which is an example of virus–virus intercellular behavior^17^. We are prompted to write this review by a more recently discovered phage communication mechanism, the arbitrium system, first described in *Bacillus subtilis*–infecting phages phi3T and SPβ^18^. These signaling circuits enable phages to regulate the lysis–lysogeny decision, which is a critical life-history switch, based on phage density and environmental cues^18,19^. Analogous in many ways to bacterial quorum-sensing (QS) systems, phage-encoded communication permits individual virions to “sense” the cumulative history of infection events and adapt their replication strategies to maximize long-term fitness at the population level^19,20^.

The three-component arbitrium module comprises: (i) AimP, a short peptide precursor secreted during infections; (ii) AimR, an intracellular receptor-transcription factor; and (iii) AimX, a non-coding RNA that downregulates lysogeny genes^18,20^. In early infection stages, low extracellular concentrations of mature AimP allow AimR to bind operator DNA and activate aimX transcription, promoting lytic replication. As AimP accumulates with successive infection cycles, its binding to AimR allosterically inhibits DNA binding, leading to repression of aimX and a shift toward lysogeny^18,20^.

Structural analyses have elucidated the mechanistic underpinnings of arbitrium-mediated regulation. High-resolution structures of SPβ AimR reveal that the mature AimP peptide docks within a C-terminal tetratricopeptide repeat (TPR) domain, inducing conformational rearrangements that disrupt the N-terminal helix–turn–helix DNA-binding module^21^. Complementary biophysical studies demonstrate that AimR exists as a dynamic dimer adopting preformed conformations optimized for either peptide binding or operator recognition, ensuring precise control over the lysis–lysogeny switch^22^.

Beyond *Bacillus* SPβ–like phages, peptide-based communication modules are pervasive among temperate phages infecting soil and pathogenic Gram-positive bacteria. Metagenomic surveys have uncovered a broad repertoire of arbitrium-like loci in diverse *Bacillus* species, each encoding distinct AimP peptides and cognate receptors, indicative of rapid diversification and co-evolution of signaling codes to maintain phage specificity^19^. Such prevalence shows the ecological importance of phage communication in natural microbial ecosystems.

In addition to the arbitrium system, *Bacillus* phage phi3T encodes a second QS circuit, Rapφ–Phrφ, derived from bacterial Rap–Phr modules, which likely modulates host-derived defense pathways during infection^23^. Moreover, several phages intercept host-encoded QS signals such as *Vibrio* autoinducers to fine-tune prophage induction and lysogeny decisions in response to bacterial community dynamics^24^. These findings reveal that phage communication extends beyond self-produced peptides to encompass the integration of host signaling networks, reflecting a sophisticated multilevel evolutionary strategy for viral adaptation.

In this review, we adopt the following working definition of phage communication: the exchange and integration of molecular signals by bacteriophages to modulate their replication strategies and collective behaviors, including the lysis–lysogeny decision, in response to ecological and population-level cues. Although the archetypal peptide-based arbitrium system has been characterized in *Bacillus* phages, the broader concept includes any phage-encoded or phage-intercepted signaling pathway that enables viruses to “sense” and react to environmental parameters, similar to bacterial environmental sensor-response pathways that sense and respond to such things as QS or chemotaxis^25–27^.

For clarity, we distinguish among three related but non-identical processes discussed in this review. First, true phage communication refers to signal-mediated coordination in which phages produce, accumulate, and detect dedicated signals that alter downstream phage behavior^18,19,28^. Second, host-signal interception refers to cases in which phages exploit bacterial communication pathways, such as QS, as cues for infection decisions^29–32^. Third, broader environmental cue integration refers to phage responses to physiological context, such as host stress, nutrient status, or infection conditions, without necessarily involving exchange of a dedicated phage-derived signal^17,30,33^. We make these distinctions because the underlying mechanisms, evolutionary interpretations, and levels of experimental support are not equivalent, even when the outcomes converge on similar life-history decisions. This distinction is particularly important in biofilms, where spatial structure, diffusion limitation, matrix adsorption or retention, and heterogeneous local multiplicity of infection (MOI) can reshape both infection dynamics and the biological meaning of these signals^34–36^.

The objectives of this review are threefold. First, we survey the molecular diversity and mechanistic architectures of known phage communication modules including peptide-mediated, small-molecule, and host-signal integration systems across various viral lineages. Drawing on metaviromic and genomic surveys, we highlight how elaborate signaling repertoires are not confined to double-stranded DNA phages but may also extend into the underexplored realms of single-stranded DNA and RNA phages^37^. Second, we critically examine the eco-evolutionary drivers that have shaped the emergence and maintenance of communication strategies in phages. By integrating resource-competition models, host-density-dependent fitness landscapes, and multilevel selection frameworks, we discuss how communication can optimize lytic versus lysogenic outcomes in fluctuating communities^26^. Third, we evaluate the translational potential of phage communication systems for therapeutic innovation. We consider how engineered signaling circuits might be deployed to improve the specificity, efficacy, and safety of phage-based interventions against multidrug-resistant bacterial pathogens^38^.

Structured searches were conducted in PubMed, Web of Science, and Scopus (2000–2024) using combinations of terms including “bacteriophage,” “phage,” “communication,” “quorum sensing,” “arbitrium,” “lysis-lysogeny,” and “signaling.” Forward citation tracking of key foundational papers and manual reference list screening supplemented the database search. Inclusion criteria: (i) peer-reviewed English-language articles; (ii) direct investigation of phage-encoded or phage-intercepted signaling mechanisms (peptide-based, small-molecule, or host-signal eavesdropping); (iii) ecological, evolutionary, or modeling studies of phage communication; (iv) translational work manipulating or evaluating phage signaling circuits. Exclusion criteria: studies of bacterial QS without demonstrated phage involvement; general phage biology without communication focus; non-English articles and preprints; eukaryotic viruses except for direct comparison. Evidence tiering: Tier 1 (established mechanisms)—validated molecular characterization including structural data (e.g., SPβ AimR-AimP). Tier 2 (strongly supported)—consistent genetic/phenotypic evidence from multiple studies (e.g., DMS3 Aqs1). Tier 3 (emerging/hypothetical)—suggestive genomic or preliminary evidence requiring validation (explicitly noted as such). Scope boundaries: We intentionally excluded detailed phage structural biology unrelated to signaling; comprehensive therapy resistance mechanisms except where communication-linked; bacterial CRISPR-Cas without phage-encoded counter-signaling. Metagenomic surveys are cited to indicate potential diversity, with appropriate caveats. Conflicting evidence is presented with the current weight of evidence, and knowledge gaps are explicitly identified in the section “Current challenges and knowledge gaps.” This framework enables readers to assess completeness and potential selection biases in our narrative integration.

Collectively, this review aims to provide a coherent, multilevel perspective on phage communication, bridging detailed molecular insights with population-level dynamics and practical applications and to delineate outstanding questions and opportunities at the interface of viral signaling, ecology, evolution, and medicine.

Integrating molecular insights into phage communication with evolutionary theory and therapeutic development is essential for a comprehensive understanding of viral strategies and for harnessing phages as precision antimicrobials. At the molecular level, detailed characterization of phage-encoded effectors that interfere with bacterial QS reveals how viruses co-opt host regulatory networks to optimize infection. For example, the *P. aeruginosa* phage DMS3 produces small protein Aqs1, which binds LasR transcription factor to block QS-controlled anti-phage defenses, enhancing phage replication^39^. Likewise, Pf family bacteriophages encode PfsE to inhibit *P. aeruginosa’s* Pseudomonas Quinolone Signal (PQS) signaling pathway, reducing host resistance and promoting viral propagation^40^. In Gram-positive species, streptococcal phages secrete the protein Paratox to antagonize the regulator ComR, demonstrating convergent evolution of QS-targeting strategies across distinct phage lineages^41^.

From an evolutionary perspective, phage communication modules ranging from peptide-based arbitrium systems to QS interception circuits have arisen under selective pressures that favor context-dependent life-cycle decisions. QS-mediated control of the lysis–lysogeny switch in *E. coli* phage T1 illustrates how host population density and metabolic state guide optimal viral strategies^42^. In soil microbiomes, bacterial autoinducers can trigger prophage induction events, synchronizing viral life cycles with host community dynamics and altering ecosystem-level bacterial composition. A review of QS in phage–host interactions underscores that these signaling networks are maintained by co-evolutionary feedback, shaping phage host range, signal specificity, and the stability of communication loci in viral genomes^43^.

Translationally, bridging molecular and evolutionary knowledge informs rational phage therapy design. Elucidating the molecular basis of phage resistance in clinical pathogens, such as cell-surface modifications conferring resistance in *Enterococcus faecalis*, will guide the selection or engineering of phages capable of overcoming such defenses^44^. Further, therapeutic efficacy can be enhanced by combining phages with QS modulators, which is further detailed below^45,46^. Finally, phage training, which leverages adaptive dynamics to target receptor usage and circumvent bacterial escape, represents a promising strategy for durable antimicrobial interventions^47^.

By uniting detailed molecular mechanisms with eco-evolutionary frameworks and clinical applications, researchers may in future be able to develop next-generation phage therapies that are both mechanistically informed and evolutionarily robust.

## Molecular mechanisms of phage communication

The arbitrium communication system was first uncovered in *Bacillus subtilis*–infecting phages phi3T and SPβ, revealing a peptide-based signaling circuit that enables temperate phages to coordinate their lysis–lysogeny decisions according to phage population density^48^. This system comprises three genes: aimP, encoding the precursor of the arbitrium peptide; aimR, encoding a peptide-responsive transcription factor; and aimX, encoding a small regulatory RNA that promotes the lytic cycle by antagonizing lysogeny functions^28,48^. Upon infection, AimP is synthesized as a propeptide, secreted into the extracellular milieu, and processed into a mature hexapeptide that accumulates with successive rounds of infection^24,48^. In early infections, low extracellular AimP concentrations permit AimR to adopt a DNA-binding conformation, activating aimX transcription and driving lytic replication^24,28^.

As AimP accumulates, it is internalized by infected cells and binds AimR with high specificity, triggering a conformational switch that abolishes the transcription factor’s DNA-binding affinity^28^. Structural studies of AimR from SPβ revealed that peptide docking into the C-terminal tetratricopeptide repeat domain induces reorientation of the N-terminal helix–turn–helix motif, preventing aimX activation and favoring lysogeny^28^. Biophysical analyses further demonstrated that AimR exists as a dynamic dimer, sampling preformed peptide-bound and DNA-bound conformations, thereby ensuring a sharp and reversible switch between life-history strategies in response to small changes in AimP concentration^28^. Comparative genomics and metaviromic surveys indicate that arbitrium-like systems are widespread and abundant among temperate phages infecting Bacillales such as *Bacillus anthracis*, *Bacillus cereus*, and *Bacillus thuringiensis*, each utilizing distinct AimP peptide sequences and cognate AimR receptors, underscoring rapid co-evolution of signaling specificity^24,48^.

**Fig. 1:**
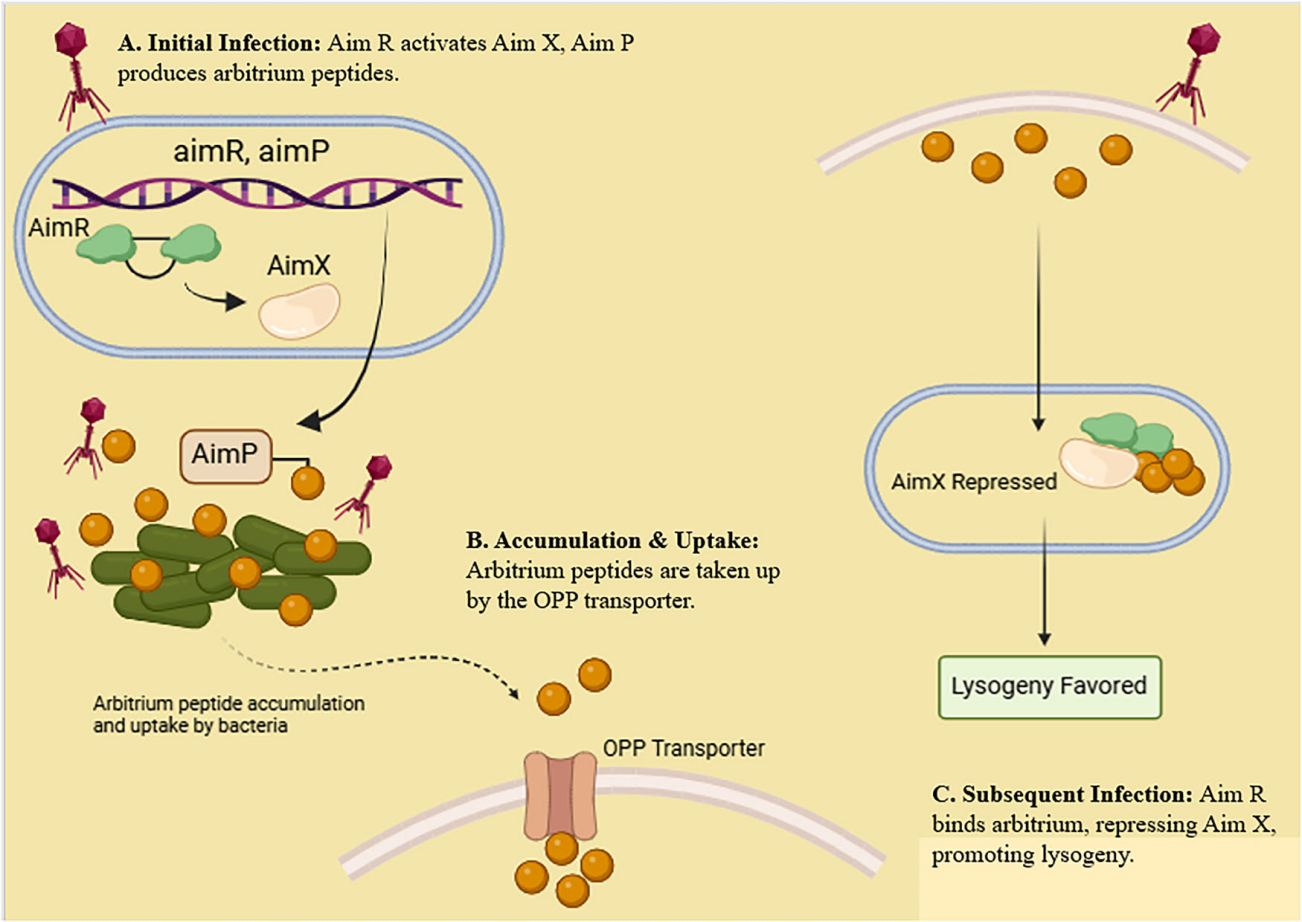
Mechanistic model for communication-based lysis-lysogeny decisions. **A** Dynamics of arbitrium accumulation during infection of a bacterial culture by phage. Upon initial encounter with a bacterial population, the early genes Aim R and Aim P are promptly expressed following infection. Aim R, functioning as a dimer, activates the expression of Aim X, an inhibitor of lysogeny. This inhibition is potentially mediated by Aim X, a regulatory non-coding RNA, directing the phage towards a lytic cycle. Simultaneously, Aim P is expressed, secreted, and processed extracellularly to generate the mature arbitrium peptides (represented as yellow spheres). **B** At later stages of infection, the arbitrium peptide accumulates in the medium and is subsequently internalized into the bacteria by the oligopeptide permease system (OPP) transporter. **C** Upon subsequent phage infection of the bacterium, the expressed Aim R receptor binds the arbitrium molecules, preventing their activation of Aim X expression. Consequently, lysogeny is favored.

At the heart of the arbitrium system is the antagonistic regulation of aimX by AimR: in the absence of peptide, AimR binds upstream of the aimX promoter to drive expression of the noncoding RNA, which inhibits lysogeny genes and commits the phage to lysis. Conversely, peptide-bound AimR cannot occupy the operator, resulting in repression of aimX and a switch to lysogenic integration^28,48^. Deletion of aimP in lysogens leads to increased spontaneous prophage induction, indicating that host-encoded peptide production in the lysogenic state feeds back into the system to stabilize dormancy until signaling thresholds for induction are met (Fig. 1)^24^.

Mathematical modeling supports the notion that arbitrium confers a density-dependent fitness advantage: by switching to lysogeny when phage progeny are abundant, temperate phages avoid depleting their host population, thereby maximizing long-term propagation opportunities. The integration of peptide signaling with canonical SOS-mediated induction pathways further allows prophages to gauge host-stress signals and phage-density cues, orchestrating induction only when environmental and population parameters align for successful vertical and horizontal transmission^24^. Collectively, the discovery and mechanistic dissection of the AimP–AimR–AimX pathway exemplifies how phages have evolved sophisticated molecular communication systems to regulate life-history transitions in a social context.

Beyond the SPβ-like arbitrium circuits, multiple phages have evolved peptide-based systems that resemble bacterial QS to coordinate life-cycle decisions. Metagenomic surveys of Bacilli-infecting phages have uncovered numerous distinct aimP–aimR–aimX-type loci, each encoding a unique hexapeptide signal and cognate transcriptional receptor, indicative of rapid co-evolution and code diversification for phage-specific signaling^19^. In *Bacillus* phage phi3T, a second peptide circuit Rapφ–Phrφ derives from bacterial cell-cell communication mechanism Rap–Phr modules and is thought to delay public-good production by newly lysogenized hosts, optimizing host and prophage fitness under high phage loads^23^. Even canonical lytic phages can exhibit interviral communication. For instance, as mentioned above, T-even phage family phenomenon of LIN represents a well-characterized example of virus–virus communication, and while this signal is not peptide-based or related to QS, it acts as a proxy for local phage density due to competition for the same host cell^17^.

Although direct evidence remains limited, recent analyses suggest that phages may also employ noncoding RNAs and CRISPR-Cas components for intra- and inter-viral signaling. Prophage loci have been described that co-encode small predicted RNAs alongside putative CRISPR arrays and ancillary Cas genes, indicating that some phages may utilize RNA cues or deploy phage-borne CRISPR systems to modulate competitor phages or host immunity during co-infection^49^. These small RNAs could act analogously to bacterial small non-coding RNAs binding to phage or host messenger RNAs to fine-tune expression of replication or immunity genes and may represent a cryptic layer of phage social communication awaiting further functional characterization^50,51^.

Phages often integrate host-derived chemical and physiological signals to optimally time the lysis–lysogeny switch. In *Vibrio cholerae*, for example, autoinducer molecules produced by the bacterial population have been shown to trigger prophage induction, synchronizing phage entry into the lytic cycle with high host densities^19^. Similarly, the Rapφ–Phrφ module in phi3T may sense nutrient-limitation signals transduced via Rap histidine kinases to delay lysis until host physiological conditions recover^23^. Classical temperate phages such as λ also exploit the host SOS response: DNA damage activates RecA, which promotes autocleavage of the CI repressor, allowing prophages to enter lytic replication in response to host stress^17^. Collectively, these systems show that phage communication extends beyond viral self-signals to encompass environmental and host-derived cues, enabling viruses to make context-dependent decisions that maximize their long-term fitness^52,53^.

The discovery of coliphage Lambda in the early 1950s by Esther Lederberg^54^ led to an explosion in knowledge around phage communication. Phage communication in Lambda resembles the arbitirium system in that both use molecular switches, responding to ecological conditions, to regulate lysis versus lysogeny, while optimizing long-term phage survival and avoiding host populations. There are, however, major differences; for example, the lambda system is an intracellular, single-cell decision-making framework that measures the condition of the host cell, and if the cell is stressed or infected by multiple phages, lysogeny is favored^55^. While the arbitrium system enables communication between virions, there is no quorum system known in Lambda. Lambda, with its system of cl and Cro repressor proteins, favoring lysogeny and lysis respectively, became the prototype for the idea of a genetic switch. Examination of the molecular switches in phage Lambda laid the foundation for modern molecular biology.

Temperate and lytic phages frequently intercept or modulate host QS pathways to optimize their life-cycle outcomes. As previously mentioned, in *E. coli*, phage T1 was shown to integrate host metabolic state and QS signals via the cAMP receptor protein (CRP), to influence prophage induction in response to population density and nutrient status^42^. Similarly, in *V. cholerae*, the autoinducers AI-2 and CAI-1 produced by the bacterial community can directly induce prophage activation, ensuring lytic replication occurs primarily when host density is high, thus maximizing dissemination^45^.

In contrast, phages of *P. aeruginosa* provide notable examples of phage-encoded QS antagonists. Phage DMS3 expresses Aqs1, a small protein that binds the LasR transcription factor, effectively suppressing QS-controlled anti-phage defenses (e.g., CRISPR-Cas systems, restriction–modification systems, and oxidative stress responses) while enhancing phage productivity^39^. Pf prophages likewise secrete PfsE to disrupt the PQS pathway and inhibit type IV pili, dismantling host communal immunity and preventing superinfection by competing phages^40,56^.

These observations illustrate a bidirectional “eavesdropping” and “jamming” model whereby phages detect host-derived QS cues to inform lysis–lysogeny decisions and, in turn, release effectors to suppress host-driven community defenses. From a therapeutic standpoint, the role of QS in inhibiting predation by phages in some human pathogens suggests that co-administration of quorum-quenching compounds during phage therapy may help maintain phage infectivity in biofilm infections^34,45,57^. However, caution is warranted, as QS inhibitors can result in upregulation of virulence factor expression in pathogens such as *V. cholerae*^45,58^.

Superinfection exclusion (Sie) mechanisms enable a resident prophage to protect its lysogenized host from subsequent infections by the same or closely related phages. Sie is typically mediated by prophage-encoded proteins that obstruct adsorption, DNA injection, or early gene expression of superinfecting virions^59^. An example of Sie is in *Lactococcus lactis*, where the temperate phages LC3, TP901-1, and Dub35A each encode immunity repressors and exclusion proteins that confer both homologous and heterologous resistance across multiple phage types^59^.

In *P. aeruginosa*, newly identified Sie proteins include Tip (from phage D3112) and Pf4-encoded PfsE, which bind and inhibit the PilB ATPase, suppressing twitching motility and preventing the entry of competing phages via type IV pili^56^. Further transcriptomic analyses reveal that prophages can co-opt host QS circuits to regulate their own Sie gene expression; specifically, prophage-encoded repressors are upregulated at high cell density through LasR and RhlR, ensuring optimal protection when phage encounters are most likely.

T-even phage lysis inhibition is an example of competitive signaling akin to bacterial QS. These behaviors demonstrate that virus–virus communication spans molecular peptide signals (e.g., arbitrium) to competition-driven modulation of infection dynamics^19^.

Together, these examples illustrate that phages not only “communicate” among themselves through specialized peptides and superinfection factors but also “listen” to bacterial conversations, integrating multiple signals to finely tune their replication strategies within complex microbial communities^18,60^.

Phage-encoded communication systems confer fitness benefits by allowing viruses to tailor their reproductive strategies to both viral and host population parameters. Rather than relying on hard-wired, single-strategy programs, communicating phages can monitor the concentration of self-generated or host-derived signals to decide whether to enter lytic replication^19^ (maximizing immediate progeny) or lysogeny (preserving host resources and ensuring future opportunities).

The *Bacillus* arbitrium system (AimP–AimR–AimX) exemplifies density-dependent decision-making. Early in an outbreak, low AimP peptide levels permit AimR to activate aimX, committing to lysis and rapid amplification. As AimP accumulates, AimR becomes peptide-bound and silenced, triggering a switch to lysogeny, which conserves the dwindling host pool^18,61,62^.

Mathematical models of arbitrium show that phages which switch to lysogeny at high viral densities avoid “boom-and-bust” collapses of susceptible hosts and achieve higher long-term yields compared to phages lacking communication^63^. This density-dependent strategy acts as a striking example of Parrondo’s paradox, in which alternating between two individually losing strategies (unrestrained lysis or obligate lysogeny) can yield a net gain when properly timed^64^.

Some phages co-opt bacterial QS signals as proxies for host abundance. In *Vibrio* phage VP882, the host-produced autoinducer VqmA binds the phage receptor to induce lysis only when *V. cholerae* cell density is high, synchronizing phage propagation with optimal transmission conditions^32^. Likewise, *E. coli* phage T1 integrates QS-linked metabolic cues via the cAMP receptor protein to modulate prophage induction in accordance with nutrient status and cell density^42^.

By delaying lysis when host cells are scarce, communicating phages prevent the collapse of local bacterial populations, which is a form of collective restraint that maximizes overall phage fitness. Conversely, when hosts are abundant, immediate lytic growth allows rapid colonization of new niches. In mixed-strain infections, cooperation among anti-CRISPR–encoding phages displays positive density dependence: co-infecting phages donate anti-CRISPR functions to one another, which helps to overcome host immunity most effectively at high MOI^65^.

Bacterial hosts and their phages are engaged in a dynamic arms race. As phages evolve sophisticated communication modules to optimize infection, bacteria deploy counter-strategies that disrupt or hijack those signals^66^. Simultaneously, phages exchange and recombine signaling loci among themselves, leading to increasingly complex and interchangeable communication repertoires.

### Host counterstrategies

Many bacteria evade phage predation through surface modifications that alter or mask the receptors recognized by phage tail fibers. For example, *Klebsiella pneumoniae* can escape podoviruses by mutating a “backup” tail-binding site on its O-antigen polysaccharide, which selects for compensatory mutations in phage ORF59 tail proteins to restore adsorption^67^. In *Bacillus*-phage coevolution experiments, mutations in tail-spike genes enable phages to infect previously resistant hosts, while hosts remodel wall teichoic acids to block infection^68^.

Alternatively, some bacterial strains acquire novel restriction–modification systems or mutate DNA methylation patterns in response to phage-delivered anti-CRISPRs. Conversely, phages may covalently modify their genomes (e.g., glucosylation of T4 DNA) to resist host nucleases, a countermeasure that selects for host evolution of new endonuclease specificities^69^. Further, while some phages disrupt bacterial QS, bacteria can also secrete enzymes like lactonases and acylases that degrade *N*-acyl homoserine lactone signals. This undermines bacterial QS and can prevent QS-dependent prophage induction (e.g., in VP882-type phages)^45^. *Serratia grimesii* uses its LuxIR-type QS system to upregulate its Type I-E CRISPR-Cas locus at high cell density, mounting an adaptive immune response precisely when phage densities and the risk of infection are greatest^70^.

### Horizontal gene transfer (HGT) and the evolution of signaling complexity

Phage genomes are modular, with recombination hotspots near signaling loci driving the horizontal transfer of communication modules across diverse viral lineages^71^. Comparative genomics of *Bacillus*-infecting phages reveals the frequent swapping of aimP–aimR–aimX cassettes, generating bespoke peptide codes and receptor specificities that limit cross-talk among co-occurring phages^18^. Filamentous phages can acquire bacterial Rap–Phr modules, turning host signaling regulators into phage-controlled switches that delay host public-good production under prophage control^23^.

Phage satellites represent another example of HGT that employs communication systems. Phage satellites are mobile genetic elements found in bacterial genomes that can sense the internal environment of a cell, and which depend on a helper virus for their lifecycle and for HGT^72,73^. For example, phage satellite PLE in *V. cholerae* detects and sabotages infecting helper phage ICP1 and responds by activating their lifecycle, resulting in PLE exclusion from the bacterial chromosome, replication, and transduction to neighboring cells; PLE exploits phage-encoded helicases, showing it responds to phage proteins^74^. Phage satellites carry regulatory genes that communicate with helper phages and host physiology, enabling them to regulate their lifecycle^73^.

Moreover, prophage clusters embedded in *Pseudomonas* genomes often co-localize with small-RNA regulators alongside orphan QS receptors and partial CRISPR-Cas arrays, suggesting that recombination can seed novel inter-phage and phage–host regulatory circuits whose functions remain to be elucidated. Directed evolution studies confirm that recombination among related phages, not point mutation alone, most rapidly expands host range and diversifies communication systems, emphasizing the importance of horizontal gene flow in the eco-evolutionary dynamics of phage signaling^71^.

Phages seldom act as solitary predators. Instead, they form dynamic communities in which related (“kin”) and unrelated virions compete for hosts, share molecular public goods, and even police cheaters^35^.

Below, we highlight the emerging principles of phage–phage social interactions and their consequences for microbiome structure and function.

Temperate phages of the Bacillales use arbitrium-like specificity codes such as highly divergent AimP peptides and cognate AimR receptors to restrict communication with kin lineages, thus preventing “cross-talk” with noncognate phages^18,19^. This molecular kin-recognition system ensures that the costly switch to lysogeny, triggered when signal levels indicate high phage density, benefits genetically related particles.

In co-infection contexts, phages encoding anti-CRISPR proteins can rescue otherwise susceptible siblings, generating a positive density-dependent effect on infection success^65^. Likewise, many *Klebsiella* and *Acinetobacter* phages secrete capsule depolymerases that degrade the bacterial exopolysaccharide matrix, a public good that accelerates diffusion of progeny virions and increases the infectivity of subsequent kin^75–77^.

In contrast, phages lacking depolymerase genes may exploit the matrix-degrading enzymes of co-infecting relatives, reaping the benefits of biofilm penetration without paying the production cost. In some systems, bioinformatic modeling suggests this may select for regulatory circuits that couple depolymerase expression to signals of kin presence, providing a mechanism to police freeloaders^78^. In addition, lysogens frequently express exclusion proteins or mask adsorption sites to block superinfecting phages including both kin and non-kin thus monopolizing host resources. In mixed-lineage communities, prophage-encoded tail-fiber variants and superinfection immunity repressors determine the competitive hierarchy among rival phages^79,80^.

These cooperative and competitive interactions are especially consequential in biofilms, where phage behavior is constrained by spatial structure rather than approximating the well-mixed conditions assumed in many mechanistic models. In structured communities, extracellular matrix can both impede and localize phage movement, adsorption opportunities are unevenly distributed, and physiological gradients generate subpopulations that differ in susceptibility, metabolic state, and local MOI. Under these conditions, communication-related phenomena such as depolymerase production, kin-restricted signaling, superinfection exclusion, and lysis timing should be interpreted not simply as abstract social traits, but as processes embedded in a heterogeneous physical environment that shapes who encounters whom, how far signals travel, and which infections become productive. This biofilm context is therefore central to understanding how phage communication influences both collective viral behavior and downstream microbiome structure^35,36,81^.

These intertwined cooperative and competitive interactions among phages profoundly shape bacterial community assembly and ecosystem function^36^. By synchronizing lysis when host densities are high and restraining infection when hosts are scarce, kin-cooperating phages stabilize bacterial population cycles and prevent clonal sweeps, thus maintaining microbial diversity^80,82^. Cooperative coinfections raise the frequency of recombination and transduction among co-infecting phages and hosts, accelerating the spread of phage resistance and signaling loci throughout the microbiome^36^. Biofilm architecture and resilience may also be directly affected by phage activity. In particular, phage depolymerases can remodel extracellular matrices, potentially opening channels for nutrient diffusion and phage movement while altering local infection patterns within structured communities^81^.

Collectively, these findings argue that phages operate as social microbes whose fitness depends not only on host exploitation but also on finely balanced cooperation and conflict with fellow viruses^35^. Appreciating this complexity will be essential for predicting phage impacts on natural and engineered microbiomes and for designing robust phage therapies that harness or disrupt viral social networks^83^. These considerations are particularly pertinent to the rational design of phage “cocktails” and phage-antibiotic combinations to improve therapeutic efficacy.

## Phage communication in clinical therapeutic contexts

Phage-encoded signaling modules originally evolved to optimize virus-host ecology can profoundly influence the success of clinical applications^28^. Natural communication circuits can affect lytic efficacy in vivo and can be harnessed or reprogrammed to gain precise control over phage behavior in clinical settings.

For example, lysogenic phages are generally considered unsuitable for clinical phage therapy; however, some studies have begun to explore the cautious use of these phages for therapeutic applications, on the basis that the presence of temperate phages may reduce bacterial load and/or deliver therapeutic benefits in limited circumstances, such as *Clostridiodes difficile* infections^84,85^. Mathematical modeling suggests such temperate phages carrying arbitrium-type circuits (AimP-AimR-AimX) may spontaneously switch from a lytic to lysogenic program once the extracellular peptide reaches a threshold concentration^86^. This could potentially undermine treatment outcomes during therapy when high local MOI are present; however, synthetic arbitrium toggles could potentially be engineered to overcome this. Swapping AimR receptors or re-coding the AimP peptide could potentially retune the lysis-lysogeny threshold to higher peptide concentrations, effectively “raising the bar” for lysogeny during therapy.

The limited efficacy of most traditional antibiotics against frequently encountered biofilm-related infections in humans^87,88^ makes phage therapy for such infections highly attractive. However, in such clinical contexts, therapeutic performance depends not only on phage killing capacity but also on the physical and physiological structure of the infection community. In biofilms, extracellular matrix can restrict or localize phage diffusion, adsorption opportunities may be spatially uneven, and nutrient or oxygen gradients can generate subpopulations with reduced metabolic activity and altered phage susceptibility. Under these conditions, communication-related traits such as lysis timing, density-dependent switching, and host-signal responsiveness may have different therapeutic consequences than in well-mixed systems. Further, ecological safety concerns are particularly relevant in this context where delayed lysis or signal-driven lysogeny may preserve persistent bacterial subpopulations, while interventions that alter QS or matrix architecture could produce unintended effects on pathogen behavior or on surrounding microbiome structure^31,34,36,84,86^.

The ecological advantage of LIN exhibited by classic T-even phages can paradoxically slow bacterial clearance in therapy, extending the time to achieve a critical kill threshold.

Similarly, phages that detect host autoinducers (e.g., *Vibrio* phage VP882 sensing VqmA-AI) may trigger lysis only at high cell densities. Although this can synchronize phage release with favorable transmission conditions, in biofilm-associated or otherwise spatially structured infections such delayed lysis may prolong bacterial persistence, especially where matrix barriers and physiological heterogeneity already limit uniform phage access to the community^32^.

Tail-fiber engineering can potentially be paired with synthetic communication modules so that phage infectivity and hence peptide accumulation could be restricted to defined bacterial targets^89^. Yehl et al. used deep mutational scanning to remodel T3/T7 tail fibers, both expanding host range and decoupling adsorption from natural QS-mediated decision circuits^90^. Ando et al. and Mitsunaka et al. have shown that common phage chassis can be reprogrammed with novel genetic modules including communication cassettes and therapeutic payloads via cell-free assembly or CRISPR-based editing platforms^91,92^. These approaches pave the way for “plug-and-play” insertion of bespoke signaling systems into clinical phages^93^. However, whether such engineered circuits will behave predictably in biofilm infections or complex microbiome settings remains uncertain, particularly because signal diffusion, selection for anti-phage resistance or counter-defenses, and host or microbiome responses may differ substantially from simplified laboratory conditions^36,94,95^.

Phages modified with CRISPR-Cas9 or Cas3 can also deliver programmable nuclease cassettes to pathogenic bacteria^96^. Embedding these payload genes downstream of engineered QS responsive promoters may enable spatially and temporally restricted nuclease expression, for example, only upon sensing a host autoinducer or synthetic peptide administered alongside the phage^97^.

**Fig. 2:**
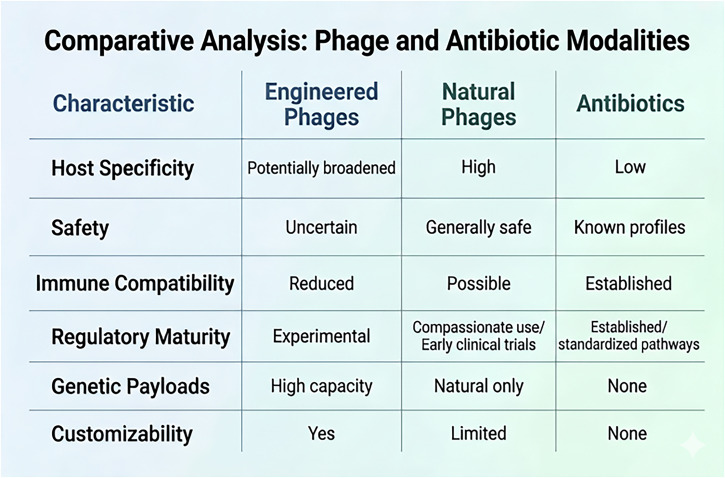
Comparative analysis of therapeutic agents: engineered phages, natural phages, and antibiotics.

By integrating engineered signaling circuits with traditional approaches to host-range expansion and immune evasion (e.g., anti-CRISPRs)^90^, next-generation phage therapeutics may be able to maintain robust lytic activity even in high bacterial load infections, avoid inadvertent lysogeny or lysis delays, be externally gated by administered small-molecule ligands, and synergize with antibiotics or immune modulators in a programmable fashion ^94^ (Fig. 2).

Harnessing and reprogramming phage communication thus offers a powerful toolkit for fine-tuning infection dynamics in vivo, maximizing bacterial clearance while minimizing the evolution of resistance^98,99^. This is both pertinent for and highly complex in the frequently encountered biofilm-related infections for which phage therapy is often sought.

There are many hurdles to be overcome in developing phages for therapy, many of which are outside the scope of this review. Microbiological examples include (i) the resistance/defense/counter-defense war between phages and bacterial targets, which has been the subject of many reviews; (ii) generating phage stocks that lack bacterial material that can trigger an immune response; such an approach would probably pair strategies around selection or engineering of bacterial host production strains to reduce immunogenicity^100^, including using cell-free systems^101^, possibly with enhanced purification of phage stocks^102^; and (iii) understanding how phages interact with the existing microbiome^103^. All of these areas deserve further intensive study.

The increasing motivation to use phage therapy for “difficult-to-treat” infections, including biofilm-related infections, necessitates a conceptual guide to compare engineered phages with natural phages and antibiotics. However, any such comparison must be interpreted cautiously, because therapeutic performance is strongly context-dependent and varies with pathogen, infection site, biofilm status, host immunity, formulation, and dosing. In particular, properties such as specificity, adaptability, safety, and resistance burden should be understood as conditional rather than absolute, especially in biofilm-associated infections and microbiome-sensitive settings.

Figure 2 outlines the core characteristics distinguishing engineered phages, natural (wild-type) phages, and conventional antibiotics in clinical applications. Engineered phages potentially provide high host specificity and genetic modularity, enabling precise design for targeted infection control. Their main challenges include immune recognition—where engineered viral proteins or delivery platforms may trigger host immune responses that limit efficacy—and the perception of uncertain safety because genetic modifications are new to regulators and clinicians. They thus face significant hurdles in regulatory approval due to their synthetic nature, even if not intrinsically unsafe. Natural phages, already present in microbial ecosystems and used under compassionate-use frameworks and early clinical trials, may strike a balance between efficacy and safety: they offer moderate host targeting with a more favorably perceived safety profile, but their capacity for payload delivery or controlled adaptation is limited. As the primary therapeutic modality for bacterial infections for almost a century, antibiotics have established clinical efficacy and regulatory maturity. However, they lack specificity, almost universally disrupt the resident microbiome, and are increasingly undermined by bacterial mechanisms of antimicrobial resistance (AMR). Together, these comparisons highlight the growing interest in phage-based interventions for AMR and biofilm-associated infections, while also underscoring the need for careful, context-specific evaluation rather than broad assumptions of superiority^104–107^.

Therapeutic phages do not act in isolation but engage in a three-way dialog with bacterial pathogens and the host immune system^95^. Communication modules, both endogenous phage signals and phage-encoded effectors that mimic or interfere with host signaling, can tip this balance, shaping the inflammatory milieu, determining phage clearance rates, and ultimately influencing treatment outcomes^108^.

Studies in acute respiratory infection models have shown that successful phage therapy often requires synergy between phage-mediated bacterial killing and a competent immune response^109^. Roach et al. showed that, in *P. aeruginosa* lung infections, neutrophil and TLR-MyD88 pathways cooperate with lytic phages to clear bacteria; in neutropenic or MyD88–/– mice, phages alone failed to control infection despite robust in vitro lysis, underscoring the necessity of an intact phage-host immune axis^109^. Complementing these in vivo data, mathematical models that incorporate finite innate capacity and density-dependent immune evasion by bacteria predict a “three-way synergism” in which phages may lower bacterial load into a range that innate effectors can eliminate, whereas neither component alone may suffice^110^.

Phage communication can further tune this synergy. Some filamentous phages deliver small RNAs that dampen host inflammatory signaling. For example, Tortuel et al. demonstrated that infection by a Pf4 phage variant reduces virulence-associated traits and modulates host responses in *P. aeruginosa* infections^111^. In contrast, lytic phages can elicit pro- and anti-inflammatory cytokines from human monocytes. Belleghem et al. observed that diverse *S. aureus* and *P. aeruginosa* phages induce interleukin (IL)-6, tumor necrosis factor-α, and IL-10 to varying degrees, suggesting that phage capsid proteins may harbor pathogen-associated molecular patterns that shape immune polarization^112,113^.

Harnessing phage communication in vivo thus opens avenues for programmable immunomodulation and mitigation of anti-phage immunity. Avenues for exploration include phage-delivered QS peptides or synthetic analogs that could be co-administered to “prime” immune cells, such as a bifunctional peptide that suppresses unwanted pro-inflammatory cytokines while simultaneously triggering phage lysis at high bacterial densities. Alternatively, one might engineer phage capsids to display “self” peptides that engage host inhibitory receptors (e.g., PD-L1 mimetics), transiently deflecting excessive inflammation in sepsis settings^114,115^.

A potential challenge in repeated or prolonged phage administration is the development of neutralizing anti-phage antibodies. Hodyra-Stefaniak et al. showed that high circulating IgG titers can rapidly clear therapeutic phages in an experimental mouse model, and that antigenic cross-reactivity among related phages may worsen this effect^116^. However, it remains uncertain whether antibody generation is predictably linked to treatment failure, since clinical outcomes do not always correlate with neutralization. In one recent human nebulized-phage trial, anti-phage neutralizing antibodies were detected in serum 10–42 days post-treatment, even though bacterial load reductions were achieved and symptomatic improvements observed, highlighting that immune responses occur but do not necessarily preclude efficacy^117,118^. Similarly, in a case series of 20 patients treated with intravenous Mycobacteriophages, serum neutralizing antibodies were identified in 8 patients, potentially contributing to lack of treatment response in 4 cases, but not consistently associated with unfavorable responses in others^119^. To mitigate immune clearance, one theoretical strategy is to map phage capsid protein epitopes (especially surface loops) and engineer them via recombination (e.g., glycosylation or loop swapping) in a manner analogous to “antigenic shift,” while preserving phage signaling or functional modules—but such modifications remain largely speculative and unproven at the clinical level. In addition, without clear evidence of contexts in which serum anti-phage neutralization is predictably linked to a detrimental effect on clinical or microbiological outcomes of phage therapy, such experiments maintain low translational priority.

Finally, phage communication circuits can potentially be harnessed to minimize the rise of phage-resistant bacterial mutants^120^. A hypothetical strategy may incorporate synthetic arbitrium modules reprogrammed to delay lysis until subpopulations of bacteria have been immunogenically primed (e.g., via phage-encoded CRISPR-Cas antimicrobials) and allow the host immune system to target escape variants. Alternatively, phages equipped with inducible anti-CRISPRs under control of host cytokine-responsive promoters could momentarily suppress bacterial defenses when phage dosing and inflammation coincide, reducing selective pressure for constitutive resistance^121^.

These potential strategies illustrate how embedding communication logic within the design of therapeutic phages can transform them from simple antimicrobials into “smart” immunotherapies dynamically coordinating bacterial killing, immune modulation, and resistance management for precision infection control^122^.

Recent advances in DNA synthesis, genome editing, and circuit design may in future allow researchers to reprogram phage signaling to maximize therapeutic benefit, combine phages rationally with small-molecule drugs, and even coordinate phage action with live probiotics. Below, we highlight two emerging paradigms^123^.

By swapping AimR receptor–peptide pairs from divergent *Bacillus* phages, investigators have retuned the threshold of lysis–lysogeny switching to require much higher peptide concentrations^20^. This modification may effectively keep therapeutic phages in a lytic mode even at high MOI^20^. Modular “reporter” circuits can drive fluorescent or colorimetric readouts in probiotic sensors, reporting pathogen arrival via engineered phage-secreted peptides^124^. Recent advances in platforms for synthetic genome development, such as cell-free genome assembly or in vivo recombineering^125^, potentially allow for the fast and efficient insertion or shuffling of communication cassettes^91^. This “plug-and-play” approach could drastically accelerate the screening of new signal–receptor pairs, host-range modules, and auxiliary effectors.

### Combined use with antibiotics or probiotics

The literature does not show a clear consensus around possible synergistic effects of phage/antibiotic combination. In *P. aeruginosa* biofilms, the combination of nebulized phage PEV20 with sub-minimum inhibitory concentration ciprofloxacin or ceftazidime significantly enhances biofilm disruption compared to either agent alone^126^. This phage–antibiotic synergy effect can be dependent on antibiotic-induced expression of phage receptors and accelerated lytic cycles^127,128^. Phages that use surface-exposed proteins of the Mex multi-drug efflux systems as binding sites are expected to drive phage resistance in their target hosts that result in antibiotic susceptibility to several classes of antibiotics^129^. In addition, some lytic phages co-administered with antibiotics may limit the development of antibiotic resistance^130^. In contrast, exposing *E. coli* to sublethal streptomycin concentrations and lytic bacteriophages together promotes resistance evolution^131^. Similarly, combinations of rifampin and the *S. aureus* phage ISP have been observed to demonstrate concentration-dependent antagonism^132^.

Designer probiotics (e.g., *Lactobacillus* strains engineered to secrete quorum-quenchers or AimP-like peptides) could also be co-administered with phages to suppress pathogen QS, potentially rendering biofilms more permeable to phage infection^124^. Merging synthetic signaling circuits with classic phage–drug combinations and live microbial “cocktail” therapeutics may enable building “smart” antimicrobial platforms in the future that dynamically sense, respond to, and shape infection environments, ushering in a new era of precision phage medicine^123^.

## Current challenges and knowledge gaps

The full taxonomic and functional diversity of communication systems beyond well-studied models like *Bacillus* phages remains largely unexplored. Metagenomic and metaviromic approaches are uncovering novel putative signaling modules, but their mechanisms and ecological relevance require extensive functional validation^133^. It is unclear how widespread sophisticated communication is across different phage families (e.g., ssDNA, RNA phages) and diverse environments.

In addition, while peptide-based signaling is becoming clearer, other potential communication modalities, such as those involving small molecules, non-coding RNAs, or even structural components, are poorly understood. Elucidating these alternative mechanisms is a key priority.

Phages likely integrate multiple cues (for example, phage density, host stress, nutrient availability, presence of competing phages). How these diverse signals are processed and integrated into a coherent decision-making output at the molecular level is a complex consideration^134^.

Furthermore, ecological studies and in vivo models need to be improved to complement the studies on phage communication that have predominantly been conducted under laboratory conditions. There is a pressing need for more in vivo studies within natural or clinically relevant environments (e.g., gastrointestinal or respiratory tract microbiomes, biofilms, soil ecosystems) to understand how these signaling systems operate and confer fitness benefits in complex, fluctuating communities^36^. Developing tools to detect and quantify signaling molecules in situ and monitor phage responses in real time within complex microbial consortia is a significant methodological challenge. This may involve novel biosensors, advanced imaging techniques, and single-cell approaches^135^.

Integrating experimental data into more sophisticated mathematical and computational models will be essential to predict the outcomes of phage communication in diverse ecological scenarios and to understand its role in shaping microbiome structure and function.

While reprogramming phage signaling holds immense promise, ensuring the stability, specificity, and predictable behavior of engineered phages in complex in vivo environments is challenging. Further, bacteria will inevitably evolve resistance to engineered phages or strategies to disrupt their communication. Understanding these co-evolutionary dynamics is vital for designing durable therapeutic interventions.

For therapeutic applications, efficient delivery of phages and their signaling modulators to infection sites is key. Understanding their pharmacokinetics (PK) and pharmacodynamics (PD), remains a significant hurdle^136^ given the multi-directional relationships between phage PK and PD, which differs from the way small molecule PK/PD is traditionally considered^137^.

Finally, the use of genetically modified phages, especially those with engineered communication systems capable of influencing microbial communities, raises regulatory questions that need proactive discussion and adequate frameworks to guide public perception and acceptance^138^.

Addressing these challenges will not only deepen our fundamental understanding of virology and microbial ecology but also unlock the full potential of phage communication for innovative applications in medicine, biotechnology, and microbiome engineering.

Phage communication offers a unique system to study broader principles of social evolution, kin selection, cooperation, and conflict in microbial populations. Continued structural elucidation of communication components (receptors, signaling molecules, regulatory complexes) will provide crucial mechanistic insights for rational engineering.

Multi-omics integration combining genomics, transcriptomics, proteomics, and metabolomics will provide a more holistic view of phage communication networks and their interaction with host cellular machinery. Utilizing artificial intelligence and machine learning to mine large-scale sequence data for novel communication systems will also help to predict signal–receptor interactions and model complex ecological dynamics.

## Conclusion

Insights into phage communication represent a paradigm shift in our understanding of bacteriophages, revealing them as complex entities capable of collective action and nuanced environmental sensing. This review has described the multilevel nature of this phenomenon, bridging detailed molecular mechanisms, such as the elegant peptide-based arbitrium system, with their profound ecological roles and evolutionary implications. We have seen how phages deploy diverse signaling strategies to make critical life-cycle decisions, engage in intricate molecular crosstalk with their bacterial hosts and other viruses, and navigate complex co-evolutionary landscapes. The ability of phages to “listen” and “talk” not only optimizes their own fitness within dynamic microbial communities but also significantly impacts bacterial population dynamics, diversity, and ecosystem functions. These effects are likely to be especially consequential in biofilms, where spatial structure, matrix retention, and physiological heterogeneity can reshape both phage signaling and infection outcomes in ways that differ fundamentally from well-mixed environments.

The integration of molecular biology, evolutionary theory, and ecological principles provides a powerful framework for dissecting these viral conversations. Further, the burgeoning field of synthetic biology offers exciting prospects for harnessing and reprogramming phage communication pathways. This opens avenues for developing next-generation phage therapeutics that may be more precise, effective, and adaptable, capable of overcoming antibiotic resistance and modulating host-pathogen-immune interactions. The design of “smart” phages, engineered to respond to specific cues or deliver targeted payloads, exemplifies the translational potential stemming from a deeper understanding of viral signaling.

While many questions remain, particularly concerning diversity, universality, and in vivo relevance of various communication systems, the study of phage communication is poised for significant advancements. Future research will undoubtedly uncover new signaling modalities, reveal more intricate regulatory networks, and refine our ability to manipulate these systems for beneficial applications. Ultimately, deciphering the language of phages will not only enrich our knowledge of the virosphere but also provide innovative tools to address pressing challenges in medicine and microbiome engineering, solidifying phage communication as a cornerstone of modern virology and microbial ecology.

## Highlights

Bacteriophage communication is a crucial multilevel evolutionary strategy where viruses utilize molecular signals, like the peptide-based arbitrium system, to coordinate complex behaviors such as lysis-lysogeny decisions in response to population density.

This review explores the diverse molecular mechanisms of phage communication, its significant ecological impacts on microbial communities, and the intricate co-evolutionary dynamics between phages, their bacterial hosts, and other viral entities, including interactions with host QS.

Understanding and engineering these sophisticated phage signaling circuits offers substantial clinical therapeutic potential, paving the way for “smart” designer phages in the future to combat multidrug-resistant bacteria and modulate host-pathogen interactions. By integrating molecular, ecological, and therapeutic perspectives, this work underscores the complexity of phage signaling and highlights its profound implications for advancing microbiology, biotechnology, and the precise engineering of microbiomes.

## Data Availability

No datasets were generated during the current study. The following datasets were searched during the study: PubMed, Web of Science, and Scopus.
